# Plankton and marine aggregates as transmission vectors for *V. aestuarianus* 02/041 infecting the pacific oyster *Crassostrea gigas*

**DOI:** 10.1111/1758-2229.13206

**Published:** 2023-09-30

**Authors:** Lapo Doni, Giovanni Tassistro, Caterina Oliveri, Teresa Balbi, Manon Auguste, Alberto Pallavicini, Laura Canesi, Carla Pruzzo, Luigi Vezzulli

**Affiliations:** ^1^ Department of Earth, Environmental and Life Sciences (DISTAV) University of Genoa Genoa Italy; ^2^ National Biodiversity Future Center Palermo Italy; ^3^ Department of Life Sciences University of Trieste Trieste Italy; ^4^ Stazione Zoologica Anton Dohrn Napoli Italy

## Abstract

*Vibrio aestuarianus* is a bacterium related to mass mortality outbreaks of the Pacific oyster, *Crassostrea gigas* in Europe. In this study, the role of different planktonic substrates (phytoplankton cells, marine aggregates and chitin fragments) in mediating *V. aestuarianus* 02/041 infection of oysters was evaluated by controlled infection experiments. It was shown that phytoplankton cells and, to a greater extent, marine aggregates, significantly promote *V. aestuarianus* 02/041 intake by *C. gigas* maintained under stressful conditions in the laboratory. Such intake is associated with higher concentration of the pathogen in the bivalve hemolymph and compromised health status of infected oysters. In contrast, chitin particles do not play a significant role as transmission vector for *V. aestuarianus* 02/041 infecting its bivalve host. Interestingly, incorporation into marine aggregates foster extracellular proteases (ECPs) activity and a higher expression of bacterial virulence genes, that are potentially involved in bivalve infection. Results from this study contribute to elucidate transmission patterns of *V. aestuarianus* 02/041 to *C. gigas* that may be useful for the development of efficient measures to prevent and control oyster disease outbreaks.

## Graphical abstract

Using controlled infection experiments this study provides evidence that incorporation into marine planktonic substrates (i.e., phytoplankton cells and, to a greater extent marine aggregates) significantly promote intake of the pathogenic bacterial strain *V. aestuarianus* 02/041 by the Pacific oyster *Crassostrea gigas* maintained under stressful conditions in the laboratory resulting in a compromised health status of the infected animals.

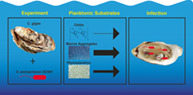

## INTRODUCTION

Oysters are filter‐feeding bivalve molluscs that inhabit estuarine and coastal environments. They include different species many of which are farmed for human consumption (e.g., *Crassostrea gigas*, *Ostrea edulis*, *Crassostrea virginica*). Oyster aquaculture represents an expanding sector worldwide with both economic and social relevance; the global production of these bivalves is about 4.8 million tonnes per year with *C. gigas* being the dominant cultured species (King et al., 2019; Pawiro, 2010).

Oyster farming, relying directly upon natural environments and feeding resources, is vulnerable to the adverse impacts of environmental conditions and infectious agents that may affect all stages of the production process. Over recent years, *C. gigas* farming industry has suffered dramatic production losses in Europe and other countries due to recurrent events of mass mortality occurring mainly in summer (Berthelin et al., 2000; Destoumieux‐Garzón et al., 2020; Malham et al., 2009). These summer mortalities episodes have been attributed to several complex causes including infectious agents, such as ostreid herpesvirus OsHV‐1 and vibrios of the Splendidus clade in spat and juveniles, and *Vibrio aestuarianus* in adults (Lupo et al., 2020). The incidence of adult oyster mortality events linked to *V. aestuarianus* has experienced a worrying increase since 2012 (Lupo et al., 2019), reaching a cumulative mortality rate of ~30% at the end of the farming cycle (Garcia et al., 2014; Parizadeh et al., 2018). The ability of virulent *V. aestuarianus* bacteria to cause disease in adult oysters is supposed to be linked to a number of secreted extracellular products, including a zinc‐dependent metalloprotease called Vam (for *V. aestuarianus* metalloprotease) (Labreuche et al., 2010; Parizadeh et al., 2018; Travers et al., 2015; Xu et al., 2018). Besides being isolated in diseased and moribund oysters, *V. aestuarianus* can also be found in coastal water as free‐living forms, in marine sediments and associated with fishes and different environmental substrates (e.g., plankton organisms, marine aggregates, chitin particles), showing a seasonal trend correlated mainly with temperature (Azandégbé et al., 2010; Vezzulli, Pezzati, et al., 2015; Vezzulli, Stauder, et al., 2015). Sediment, particulate matter and planktonic organisms also represent environmental reservoirs for *V. aestuarianus* that, as previously shown for *Vibrio cholerae* and other vibrios (Doni et al., 2023; Huq et al., 2005; Pruzzo et al., 2008), can survive in these compartments for long time, possibly forming biofilms, also during unfavourable environmental conditions (Vezzulli, Pezzati, et al., 2015; Vezzulli, Stauder, et al., 2015).

The finding that members of the plankton community and suspended particulate matter can accumulate large amounts of *V. aestuarianus* suggests that they may also act as vehicles that move these bacteria through the water column and facilitate their uptake by bivalves. Bivalves feed by pumping large volumes of ambient water and filtering suspended particles through their gills. Filtering efficiency varies with particle size; in the case of *C. gigas*, the optimal dimensional absorption is between 5 and 7 μm in diameter (Froelich et al., 2013; Kramer et al., 2016). Smaller particles are captured with an efficiency that decreases with decreasing particle size (Riisgård, 1988); for example, those less than 1 μm are captured with an efficiency of less than 20% (Kramer et al., 2016).

Thus, considering the size of 1 μm or less for many bacteria, including *V. aestuarianus*, it is likely that they are not efficiently captured by the bivalve gill sieve when in free‐living form. In contrast, bacterial association with various substrates present in the water column could increase their uptake by *C. gigas* and, consequently, the possibility for pathogenic bacteria to express factors affecting bivalve health. Interestingly, it was previously found that the ingestion rate of suspended polystyrene beads and *Escherichia coli* (range dimension 0.3–1.0 μm) by several species of bivalves is low, but it increases significantly when the same particles are incorporated into aggregates (Kach & Ward, 2008; Ward & Kach, 2009). Froelich et al. (2013) reported that both E and C genotypes of *Vibrio vulnificus* exhibit greater uptake by oysters when they are integrated into marine aggregates.

In this work, we conducted laboratory infection experiments to investigate whether different representative marine substrates (*Nannochloropsis gaditana* phytoplankton cells, marine aggregates and chitin fragments) might represent suitable vehicle of uptake and infection for the virulent *V. aestuarianus* strain 02/041 in the pacific oyster *C. gigas*. In addition, considering that interactions of bacteria with marine surface (e.g., phytoplankton cells, detritus) may result in a series of events that change the characteristics of both the colonized substrate and the colonizing microorganisms (Torres‐Monroy & Ullrich, 2018), we also investigated whether *V. aestuarianus* 02/041 incorporation into a representative marine substrate may affect its pathogenicity potential targeting extracellular aminopeptidase activity and differential gene expression.

The obtained results showed that *V. aestuarianus* 02/041 incorporation into phytoplankton cell and marine aggregate substrates promotes bacterial uptake by *C. gigas*. This uptake is associated with a reduction in the health status of the bivalve evaluated at the cellular level by means of the hemocyte lysosomal membrane stability (LMS) test. Data are also presented showing that incorporation into marine aggregates foster extracellular proteases (ECPs) activity by the bacteria and promote expression of virulence genes potentially involved in bivalve infection.

## RESULTS AND DISCUSSION

### Response of adult oysters to salinity stress

Infection by *Vibrio aestuarianus* (Anguillarum clade) is associated with the occurrence of mass mortality of adult Pacific oysters *Crassostrea gigas* in Europe (Travers et al., 2017). In preliminary experiments, adult oysters were exposed to the pathogenic strain *V. aestuarianus* 02/041 by immersion into artificial seawater (ASW, salinity 31 ppt) contaminated with the bacteria, either free‐living or associated with different substrates (*N. gaditana* cells, marine aggregates or chitin fragments) (see experimental procedures). Controls consisting of oysters immersed in ASW containing the substrate alone were also included. At the end of incubation, no mortality was recorded and no *V. aestuarianus* bacteria were found in oyster hemolymph by real‐time PCR. Moreover, hemocyte lysosomal membrane stability (LMS) analysis, which is considered the most sensitive biomarker of bivalve health status at the cellular level (OSPAR Commission, 2012), did not show any difference between controls and oysters challenged with *V. aestuarianus* in the presence or in the absence of the substrate (data not shown).

It is known that oyster susceptibility to infection depends on a number of factors that include pathogen properties, environmental quality, host physiology and health status (Destoumieux‐Garzón et al., 2020). In particular, molluscs exposed to environmental stressors, such as low salinity, exhibit higher susceptibility to infection than unexposed ones (Raftos et al., 2014). Thus, we hypothesized that virulence of *V. aestuarianus* toward *C. gigas* in the used immersion model of infection may be increased when bacteria infect oysters pre‐exposed to low salinity stress. To this end, oysters were transferred to jars containing 15 ppt or 5 ppt salinity ASW; although not optimal, these conditions should not induce lethal changes in bivalves (Knowles et al., 2014). After 2 h incubation, a 38% and 65% decrease in hemocyte LMS compared to control was observed in oysters immersed in 5 and 15 ppt ASW, respectively. According to international standard for bivalve hemocytes (OSPAR Commission, 2012), a reduction of less than 50% in LMS in comparison to control can be regarded as indicative of a mild and reversible stress for the bivalve. Therefore, oysters exposed to 15 ppt salinity ASW were used in further experiments.

### *Response of low salinity stressed oysters to* V. aestuarianus *02/041 exposure in the presence and absence of different marine planktonic substrates*

**FIGURE 1:**
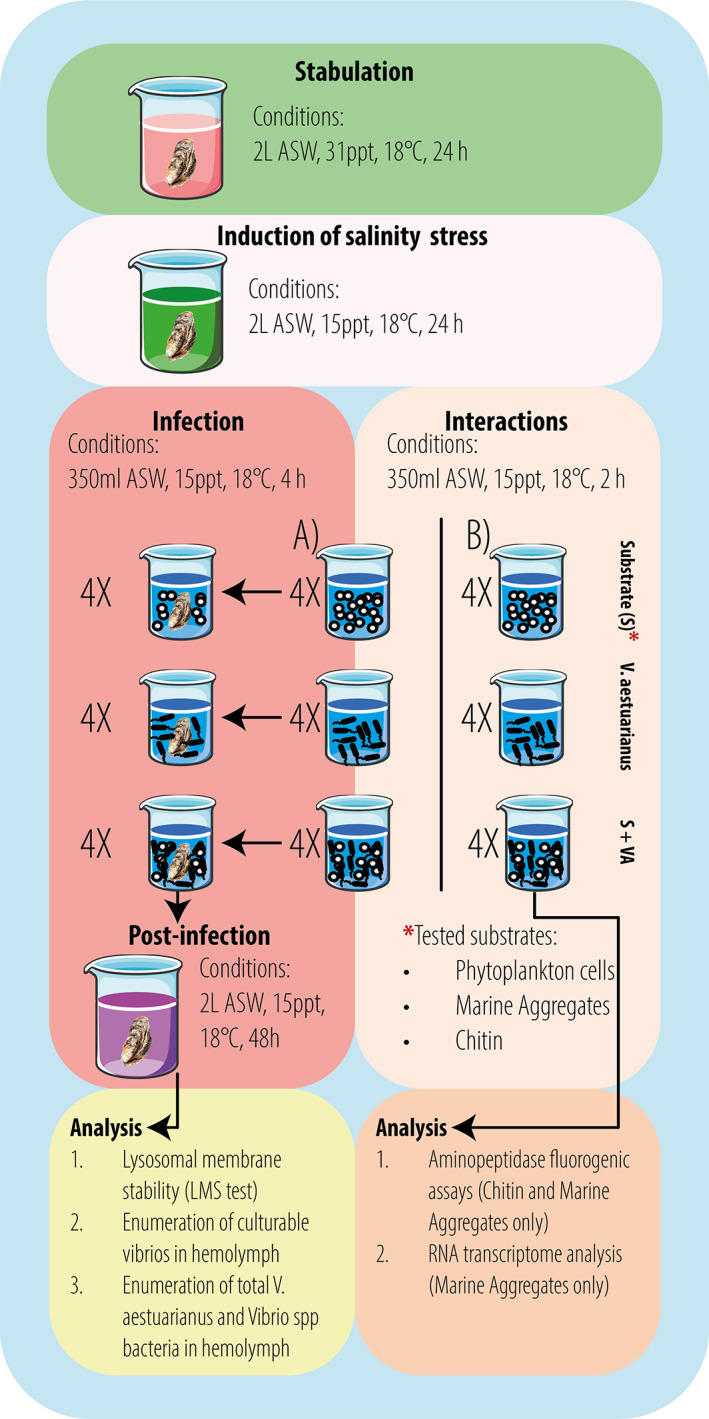
Experimental set‐up for artificial infections (A) of *Crassostrea gigas* and the study of interactions (B) of *Vibrio aestuarianus* with different marine substrates.

To assess whether marine planktonic substrates may favour *V aestuarianus* 02/041 infection of the bivalve host, individual low‐salinity stressed oysters were added to jars containing ASW (salinity 15 ppt) previously inoculated with *V. aestuarianus* 02/041, bacteria alone, the substrate alone, or mixture of bacteria plus the substrate (as an average, about 0.1% of bacterial inoculum was associated with the substrates) (Figure 1). Two days post‐infection, no oyster mortality was observed in any condition probably due to the short exposure time and low bacterial concentration (10^4^–10^5^ bact/mL) associated with the marine plankton substrates. Then, hemolymph was extracted from infected oysters to both determine concentration of total and culturable *V. aestuarianus* 02/041 and *Vibrio* spp., and evaluate hemocyte LMS. Experiments with *N. gaditana* (Table 1) and marine aggregates (Table 2) gave similar results. *V. aestuarianus* bacteria, which were absent in the hemolymph of control oysters (uninfected and challenged with the substrate alone), were detected by real‐time PCR in the hemolymph of oysters challenged with bacteria only and in those challenged with bacteria plus the substrate. Notably, in oysters exposed to bacteria plus *N. gaditana* or marine aggregates the concentration of *V. aestuarianus* was 2.3 and 15 times higher, respectively, than that of oysters challenged with bacteria only, suggesting an active role of these substrates, especially marine aggregates, in mediating *V. aestuarianus* 02/041 oyster infection. Likewise, culturable *V. aestuarianus* bacteria were found in the hemolymph of *C. gigas* infected with bacteria plus phytoplankton cells or marine aggregates at a higher concentration (1.6 and 2.9 fold increase, respectively) than in *C. gigas* infected with bacteria alone. It is noteworthy that, in both cases, about half of *V. aestuarianus* 02/041 bacteria in the hemolymph was culturable.

**TABLE 1:** *Vibrio* concentration in hemolymph and hemocyte LMS after infection experiments.

	Bacterial concentration				
	*V. aestuarianus* 02/041		Total vibrios		Hemocyte LMS (NRRT test)
Oyster exposure to	bacteria/mL	CFU/mL	bacteria/mL	CFU/mL	% control
Uninfected control	Not found	Not found	2.5 × 10^4^ ± 3.4 × 10^3^	9.3 × 10^3^ ± 6.6 × 10^2^	100 ± 1
Phytoplankton cells	Not found	Not found	2.7 × 10^4^ ± 5.4 × 10^3^	9.6 × 10^3^ ± 9.4 × 10^2^	102 ± 3
*V. aestuarianus* 02/041	**5.2 × 10** ^**3**^ **± 7.8 × 10** ^**2**^	**1.8 × 10** ^**3**^ **± 5.3 × 10** ^**2**^	**3.5 × 10** ^**4**^ **± 6.3 × 10** ^**3**^	**1.5 × 10** ^**4**^ **± 4.3 × 10** ^**3**^	**99 ± 8**
Phytoplankton cells + *V. aestuarianus* 02/041	**1.2 × 10** ^**4**^ **± 8.1 × 10** ^**3**^ *	**2.8 × 10** ^**3**^ **± 3.2 × 10** ^**2**^ *	**8.4 × 10** ^**4**^ **± 7.2 × 10** ^**3**^ *	**3.2 × 10** ^**4**^ **± 5.1 × 10** ^**3**^ *	**50 ± 8**

*Note*: Low salinity stressed oysters were exposed to either *V. aestuarianus* 02/041 bacteria, or phytoplankton (*N. gaditana*) cells, or mixture of bacteria plus phytoplankton cells. Total (bacteria/mL) and culturable (CFU/mL) bacterial concentration are showed. LMS was evaluated by the neutral red retention time (NRRT) assay; values are expressed as percent of control values (uninfected control, 100%). Reported values represent the mean of at least nine determinations ± standard deviation obtained from the three replicate experiments. Bold values are phytoplankton cells + *V. aestuarianus* 02/041 vs *V. aestuarianus* 02/041.

Abbreviation: ns, not significant.

* *p* < 0.05.

**TABLE 2:** *Vibrio* concentration in hemolymph and hemocyte LMS after infection experiments.

	Bacterial concentration				
	*V. aestuarianus* 02/041		Total vibrios		Hemocyte LMS (NRRT test)
Oyster exposure to	bacteria/mL	CFU/mL	bacteria/mL	CFU/mL	% control
Uninfected control	Not found	Not found	1.4 × 10^4^ ± 6.6 × 10^3^	9.1 × 10^3^ ± 4.7 × 10^2^	100 ± 1
Marine aggregates	Not found	Not found	1.1 × 10^4^ ± 8.4 × 10^2^	8.8 × 10^3^ ± 7.3 × 10^2^	108 ± 2
*V. aestuarianus* 02/041	**4.1 × 10** ^**3**^ **± 2.3 × 10** ^**2**^	**2.1 × 10** ^**3**^ **± 9.2 × 10** ^**2**^	**2.6 × 10** ^**4**^ **± 3.3 × 10** ^**3**^	**9.9 × 10** ^**3**^ **± 1.9 × 10** ^**3**^	90 ± 7
Marine aggregates + *V. aestuarianus* 02/041	**6.2 × 10** ^**4**^ **± 4.1 × 10** ^**3**^ *	**6.1 × 10** ^**3**^ **± 8.3 × 10** ^**2**^ *	**3.9 × 10** ^**4**^ **± 6.4 × 10** ^**3**^ *	**1.9 × 10** ^**4**^ **± 2.9 × 10** ^**3**^ *	53 ± 6

*Note*: Low salinity stressed oysters were exposed to either *V. aestuarianus* 02/041 bacteria, or marine aggregates, or mixture of bacteria plus Marine aggregates. Total (bacteria/mL) and culturable (CFU/mL) bacterial concentration are showed. LMS was evaluated by the neutral red retention time (NRRT) assay; values are expressed as percent of control values (uninfected control, 100%). Reported values represent the mean of at least nine determinations ± standard deviation obtained from the three replicate experiments. Bold values are marine aggregates + *V. aestuarianus* 02/041 vs *V. aestuarianus* 02/041.

Abbreviation: ns, not significant.

* *p* < 0.05.

The concentration of total and culturable vibrios in the hemolymph of oysters challenged with the substrate alone was similar to that of uninfected control, and increased in oysters challenged with bacteria or bacteria plus phytoplankton cells or marine aggregates. Interestingly, the increase in bacterial concentration was higher in the hemolymph of oysters challenged with the mixture of bacteria plus substrate than in the hemolymph of oysters challenged with bacteria alone. Statistically significant changes in hemocyte LMS were observed between oysters challenged with bacteria plus phytoplankton cells (Table 1) or marine aggregates (Table 2) and those challenged with bacteria alone or the substrate alone. In fact, in oysters exposed to *V. aestuarianus* 02/041 plus substrate, hemocyte lysosomal membranes were completely destabilized, indicating extremely stressful conditions, whereas no destabilization was observed in hemocytes from oysters exposed to bacteria or substrate alone.

These findings support the view that the concentration of phytoplankton cells or marine aggregates in coastal waters significantly influenced the level of oyster exposure to microbial pathogens and their associated infections.

In contrast, in experiments with chitin fragments (Table 3), no statistically significant difference was observed between the concentration of *V. aestuarianus* 02/041 (both total and culturable) in the hemolymph of low‐salinity stressed oysters challenged with bacteria alone and in the hemolymph of those challenged with bacteria and chitin. Likewise, no difference was observed in the concentration of total and culturable vibrios between oysters exposed to *V. aestuarianus* alone and those exposed to *V. aestuarianus* and chitin. Similarly, no difference in hemocyte LMS was observed between the tested samples. According to these results, although chitin substrates are considered a primary reservoir for *Vibrio* in aquatic environments (Vezzulli et al., 2010) they do not seem to represent an efficient vector for infection of the bivalve host. A possible explanation for these findings could be ascribed to chemical (e.g., carbohydrates attached to particle's surface) or physicochemical surface properties of chitin particles which, in addition to size, have been reported as important factors contributing to particle selection in oysters (Rosa et al., 2013).

**TABLE 3:** *Vibrio* concentration in hemolymph and hemocyte LMS after infection experiments.

	Bacterial concentration				
	*V. aestuarianus* 02/041		Total vibrios		Hemocyte LMS (NRRT test)
Oyster exposure	bacteria/mL	CFU/mL	bacteria/mL	CFU/mL	% control
Uninfected control	Not found	Not found	2.1 × 10^4^ ± 7.2 × 10^3^	3.1 × 10^3^ ± 8.4 × 10^2^	100 ± 1
Chitin particles	Not found	Not found	3.3 × 10^4^ ± 9.1 × 10^3^	5.1 × 10^3^ ± 9.7 × 10^2^	103 ± 2
*V. aestuarianus* 02/041	**1.9 × 10** ^**3**^ **± 7.6 × 10** ^**2**^	**7.4 × 10** ^**2**^ **± 9.2 × 10** ^**1**^	**7.0 × 10** ^**4**^ **± 8.9 × 10** ^**3**^	**2.1 × 10** ^**4**^ **± 3.4 × 10** ^**3**^	96 ± 7
Chitin particles + *V. aestuarianus* 02/041	**1.7 × 10** ^**3**^ **± 8.1 × 10** ^**2ns**^	**6.3 × 10** ^**2**^ **± 8.4 × 10** ^**1ns**^	**7.2 × 10** ^**4**^ **± 8.2 × 10** ^**3ns**^	**1.1 × 10** ^**4**^ **± 8.9 × 10** ^**3ns**^	95 ± 6

*Note*: Low salinity stressed oysters were exposed to either *V. aestuarianus* 02/041 bacteria, or chitin fragments, or mixture of bacteria plus chitin. Total (bacteria/mL) and culturable (CFU/mL) bacterial concentration are showed. LMS was evaluated by the neutral red retention time (NRRT) assay; values are expressed as percent of control values (uninfected control, 100%). Reported values represent the mean of at least nine determinations ± standard deviation obtained from the three replicate experiments. Bold values are Chitin particles + *V. aestuarianus* 02/041 vs *V. aestuarianus* 02/041.

Abbreviation: ns, not significant.

*p < 0.05.

Overall, these results show that the presence of both phytoplankton cells and marine aggregates, but not chitin fragments, increases the uptake of *V. aestuarianus* 02/041 by low salinity stressed *C. gigas*. Such higher uptake is particularly evident in the presence of marine aggregates, and it is associated with a reduction of the oyster health status. The trophic state (e.g., concentration of particulate organic matter, phytoplankton and marine aggregates) of coastal marine waters could thus be critical for the transmission dynamics of *V. aestuarianus* in oyster populations.

### *Effect of* V. aestuarianus *02/041 and* Vibrio *spp. interaction with marine planktonic substrates on bacterial extracellular protease activity*

*Vibrio* pathogenicity and virulence toward bivalves have been partially linked to the production of extracellular products (ECPs) and in particular to extracellular proteases. For example, in *V. aestuarianus*, a zinc metalloprotease (designated Vam) was identified which induces immunosuppressant activities on *C. gigas* hemocyte functions in vitro, most probably through the modulation of hemocyte cell physiology (Labreuche et al., 2010). To evaluate whether the interaction of *V. aestuarianus* 02/041 with marine aggregates and chitin fragments may stimulate extracellular protease activity, in vitro tests were performed using fluorescent markers to assess aminopeptidase activity (APA), a measure of extracellular hydrolysis of protein (Astel et al., 2018). For comparison, APA activity was also measured in other *Vibrio* strains in the presence of marine aggregates or chitin fragments. Interactions of bacterial strains with *N. gaditana* cells could not be investigated for APA being difficult to discern bacterial versus algal enzymatic activity with the used fluorimetric assay.

**FIGURE 2:**
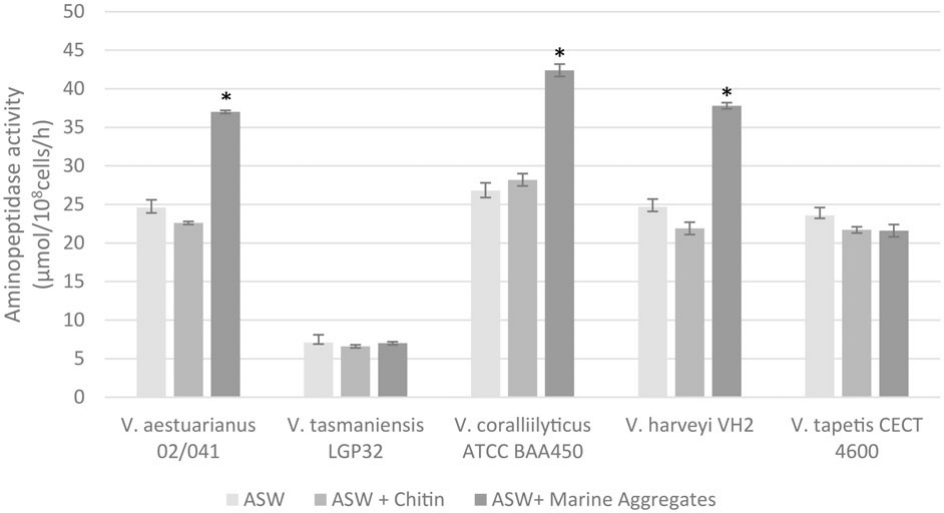
Effect of the presence of either chitin fragments or marine aggregates on extracellular aminopeptidase activity rates by *Vibrio aestuarianus* 02/041 and other different Vibrio species (*p < 0.05).

As a result, interaction with marine aggregates was observed to significantly increase APA rates in *V. aestuarianus* 02/041, *V. coralliilyticus* ATCC BAA450 and *V. harveyi* VH2 (Figure 2). In particular, *V. aestuarianus* 02/041 suspension in ASW (salinity 15 ppt) showed average APA values of 24.6 ± 0.7 μmol/10^8^ cells/h whilst in the presence of marine aggregates, APA activity increased up to 37.0 ± 0.2 μmol/10^8^ cells/h representing a 54% increase if compared with values obtained in ASW alone. In contrast, no significant increase was observed for *V. tasmaniensis* LGP32 and *V. tapetis* CECT 4600 (Figure 2). Similarly, bacterial interaction with chitin fragments did not affect protease activity rates in all the tested strains.

Interaction of *V. aestuarianus* 02/041 with marine aggregates thus increased bacterial extracellular proteases activity probably in relation to an increase of labile organic matter abundance available for heterotrophic metabolism which in turn may also play a role during host infection.

### *Transcriptome profiles of* V. aestuarianus *02/041 associated to marine aggregates*

**FIGURE 3:**
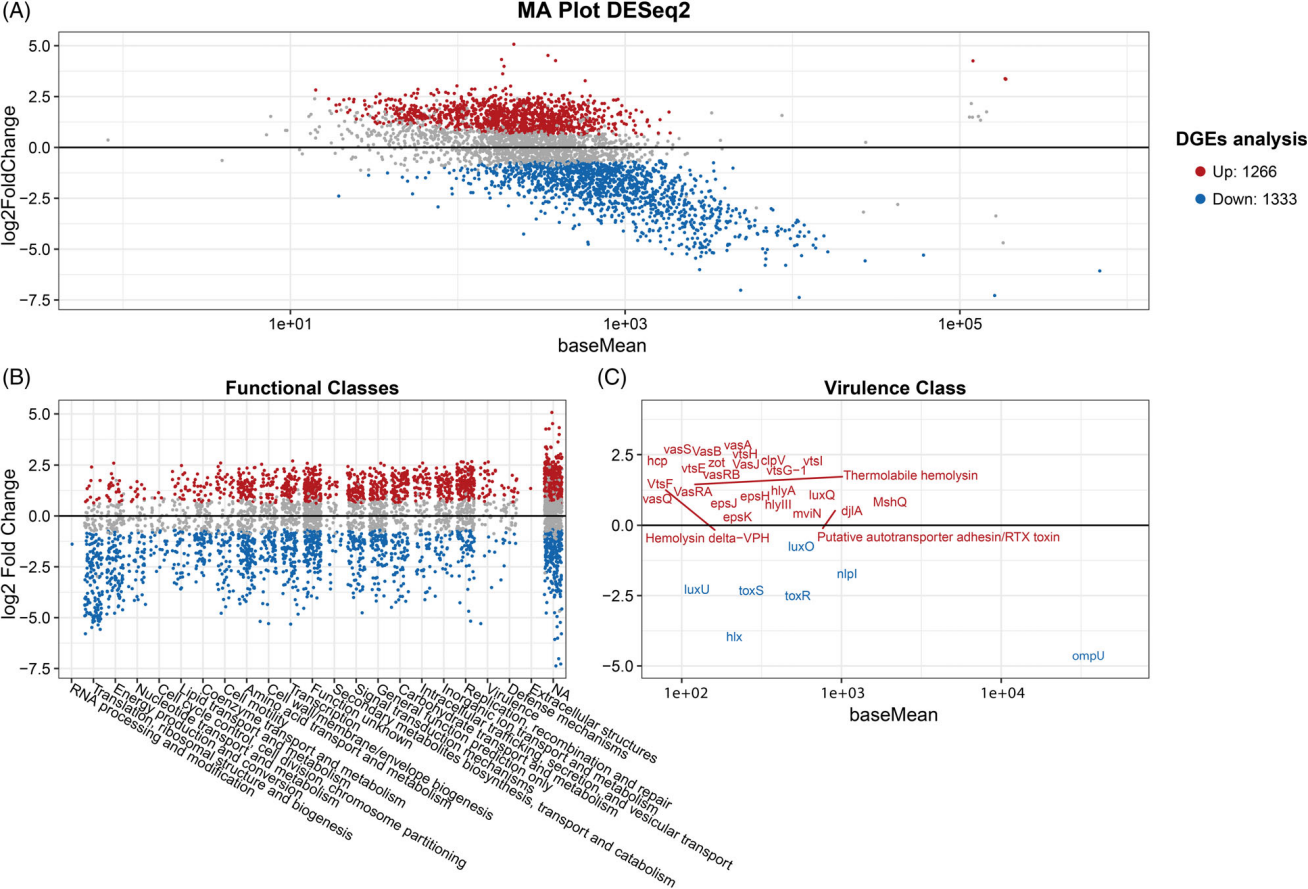
Overview of differential expression of RNA transcripts of *Vibrio aestuarianus* 02/041 associated with marine aggregates against the control (only *V. aestuarianus*). (A) MA plot displaying the log fold‐change compared with the mean expression. Coloured dots denote significantly differentially expressed genes, while grey dots denote non‐differentially expressed genes. Red dots with a positive log2 fold change indicate genes that were upregulated, and blue dots with a negative log2 fold change indicate genes that were downregulated. (B) Differential expression values for genes assigned to functional categories obtained from COG and GO. (C) MA plot displaying significantly expressed genes assigned to the virulence class.

According to above results, marine aggregates were identified as an important vehicle mediating *V. aestuarianus* 02/041 infection of *C. gigas* also stimulating ECP production by the bacteria. To further evaluate the effect of the interaction of *V. aestuarianus* 02/041 with marine aggregates on bacterial virulence, a differential transcriptome analysis of *V. aestuarianus* 02/041 associated/non‐associated to marine aggregates was performed (Table S1). Denoised reads mapped on average 85% and 15% to the first and second chromosome, respectively. A total of 4.187 differentially expressed genes (DEGs) were identified of which 1.266 genes (30%) were significantly upregulated (adjusted *p*‐value < 0.05) and 1.333 genes (32%) were significantly downregulated (adjusted *p*‐value < 0.05) in bacteria associated with marine aggregates if compared with bacteria suspended in ASW only (Figure 3, Table S1). In particular, association with marine aggregates resulted in an increase in the expression of *V. aestuarianus* genes involved in several functional pathways, including virulence, defence mechanisms, intracellular trafficking, secretion, vesicular transport, inorganic ion transport and metabolism, replication recombination and repair, signal transduction mechanisms, and carbohydrate transport and metabolism (Figure 3). Notably, several upregulated genes belonged to the virulence class and included genes encoding for type VI secretion proteins (vas and vts families) and general secretion pathway proteins (eps) (Figure 3). In addition, the virulence factor mviN, the DnaJ‐like protein membrane‐anchored DjlA, the putative autotransporter adhesin/RTX toxin, the transcriptional regulator aphA, the LysR‐type activator AphB and mshQ (a mannose‐sensitive hemagglutinin biogenesis component), hemolysin‐related genes (hlyIII, Hemolysin delta‐VPH, thermolabile hemolysin and hlyA) and the Zonular occludens toxin Zoc gene were also upregulated in the presence of marine aggregates. All these factors have been reported to play a role in bacterial virulence although their function in *V. aestuarianus* infection of *C. gigas* remain to be elucidated.

Interestingly, DEGs putatively involved in host colonization such as the tcpC gene encoding an outer membrane lipoprotein, and genes encoding for type IV pilin assembly protein and mannose‐sensitive hemagglutinin (MSHA) pilus biogenesis were also significantly upregulated in *V. aestuarianus* 02/041 cells associated to marine aggregates. Colonization is the first stage of microbial infection and require factors such as pili or outer membrane proteins mediating pathogen attachment to the surface of the host (Vezzulli et al., 2008). In particular, the MSHA pilus is known to promote *V. cholerae* attachment to bivalve hemocytes thus playing a role during infection (Stauder et al., 2012). MSHA is present in a large proportion of *V. aestuarianus* isolates and is also involved in interactions of the bacterium with environmental surfaces and *V. aestuarianus* persistence in the environment (Pezzati et al., 2015).

## CONCLUSION

Oysters filter sediment particles, detritus and small phytoplankton from brackish and marine waters. Filtration of marine substrates is crucial for oyster feeding and grow but could also serve as a vehicle for infection by microbial pathogens. Results from this study showed that phytoplankton cells and marine aggregates significantly promote *V. aestuarianus* 02/041 uptake by *C. gigas* maintained under stressful conditions in the laboratory. Such an uptake is associated with a compromised health status of infected oysters. In contrast chitin particles, previously identified as preferential reservoirs for this bacterium in the aquatic environment (Vezzulli, Pezzati, et al., 2015), did not appear to foster its transmission to the bivalve host. Phytoplankton cells and marine aggregates both constitute a food source for *C. gigas* and can be filtered efficiently by the bivalve suspension feeding apparatus. Pathogenic bacteria such as *V. aestuarianus* 02/041 might take advantage of this by adhering to food particles and entering the bivalve host more efficiently. Interaction with marine aggregates was also shown to significantly affect virulence properties of the bacterium which may thus play an additional role during infection.

Overall, routes of transmission of *V. aestuarianus* 02/041 to oysters identified in this study may be useful for the development of efficient measures to prevent and control oyster disease outbreaks.

## EXPERIMENTAL PROCEDURES

### Bacteria and culture conditions

The virulent *V. aestuarianus* strain 02/041 kindly supplied by Dr. Isabelle Arzul and Dr. Marie‐Agnès Travers (IFREMER, France) was used for the infection experiments (De Decker & Saulnier, 2011; Goudenège et al., 2015). The bacterial strains *V. tasmaniensis* LGP32, *V. coralliilyticus* ATCC BAA450, *V. harveyi* VH2 and *V. tapetis* CECT 4600 were also used in aminopeptidase fluorogenic assays. Bacteria were cultured overnight under constant shaking at 24°C in Luria‐Bertani (LB) broth (Scharlau, Italy) containing 3% NaCl. Cells were centrifuged at 4500*g* for 10 min and the resulting pellet was washed twice in PBS (0.1 M KH_2_PO_4_, 0.1 M Na_2_HPO_4_, 0.15 M NaCl, pH 7.2–7.4), and resuspended in the same buffer to the final concentration of about 1 × 10^10^ CFU/mL. Thiosulfate Citrate Bile salts Sucrose (TCBS) agar (Scharlau, Italy) was also used for isolation of *Vibrio* colonies. Filter sterilized artificial sea water (ASW) of different salinity (31 ppt, 15 ppt and 5 ppt) were used in infection experiments. *V. aestuarianus* 02/041 bacteria long term survival in ASW of different salinities (31 ppt and 15 ppt) was analysed by evaluating the number of culturable bacteria onto TCBS at timed intervals. Bacterial survival was unchanged during 3 day incubation (data not shown).

### Oyster stabulation and induction of low salinity stress

Adult *C. gigas* oysters, 8–10 cm long, originating from France (Bay of Biscay) were bought on a local market, and kept for 24 h at 18°C in static tanks containing aerated ASW (salinity 31 ppt) (2 L/oyster). In preliminary experiments salinity stress was induced by replacing ASW (salinity 31 ppt) with low‐salinity ASW (15 ppt or 5 ppt). After 2 h incubation, oyster health status was evaluated by hemocyte lysosomal membrane stability‐LMS test. Salinity of 15 ppt was selected for infection experiments (see Result section).

### Substrates

*Nannochloropsis gaditana* phytoplankton cells were cultured in Walne medium (Walne, 1966) at 25°C aerobically in the presence of light. After 7‐day incubation, exponentially growing cells were centrifuged at 4500*g* for 10 min and the resulting pellet was resuspended in ASW (salinity 15 ppt) to a final concentration of about 4–6 × 10^7^ cells/mL, as evaluated by microscopy examination (average cell diameter of 2–3 μm). Marine aggregates (Two Little Fishies Inc, Miami, USA), consisting of fragments (0.2–150 μm size) of phytoplankton (*Nannochloropsis*, *Tetraselmis*, *Isochrysis*, *Spirulina* and *Schyzochitrium*) and zooplankton organisms (as specified by manufacturer instructions), were used (approximately 4–6 × 10^7^ particles/mL, as evaluated by microscopy examination). Shrimp shell chitin (Sigma–Aldrich) was resuspended in ASW (salinity 15 ppt) and treated with a potter homogenizer. The resulting suspension was filtered on a 100 μm net in order to select particles suitable for oyster filtration (Froelich et al., 2013; Kamiyama, 2011), and sterilized by autoclave. After centrifugation at 4500*g* for 10 min, the resulting pellet was washed twice in ASW (salinity 15 ppt) and resuspended in ASW (salinity 15 ppt) to a final concentration of about 4–6 × 10^7^ chitin fragments/mL, ranging 5–100 μm particle size (as evaluated by microscopy examination). Aliquots of the potential vector suspensions were analysed for the presence of culturable and total *V. aestuarianus* (see below) to ensure that they were not infected before the experimental trials.

### *Interaction of* V. aestuarianus *02/041 bacteria with the different substrates*

Before infections *V. aestuarianus* 02/041 (final concentration about 1 × 10^8^ bacteria/mL) associated/non associated with the different substrates (final concentration about 1 × 10^5^ *N. gaditana* cells, or marine aggregates or chitin fragments per mL) were added to 350 mL ASW (salinity 15 ppt) in 0.5 L jars. Controls with the substrate alone were also included. Experiments were performed in quadruplicate (12 oysters tested per substrates) and repeated three times (Figure 1).

### Oyster infection experiments and hemolymph extraction

Oysters (both exposed and non‐exposed to low salinity stress) were challenged by immersion to *V. aestuarianus* 02/041 associated/non‐associated with the different substrates (*N. gaditana* cells or marine aggregates or chitin fragments) (Figure 1A); controls with the substrate alone were always included. Briefly, 0.5 L jars containing 350 mL ASW (salinity 15 ppt) were inoculated with either bacteria alone (final concentration about 1 × 10^8^ bacteria/mL), or substrate alone (final concentration about 1 × 10^5^ *N. gaditana* cells or marine aggregates or chitin fragments per mL), or bacteria plus substrate. Individual oyster was placed in each jar kept for 4 h at 18°C. Oysters were then removed from the jars, placed into clean 2 L jars containing fresh ASW (salinity 15 ppt) and incubated at 18°C for further 2 days. Each oyster was then removed from experimental jars, rinsed with ASW and patted dry with paper towels. Hemolymph was extracted from the posterior adductor muscle using a sterile 1 mL of syringe with an 18 G1/200 needle for LMS test, enumeration of culturable vibrios and of *Vibrio aestuarianus/Vibrio* spp. by real‐time PCR. Controls with uninfected oysters were always included to evaluate background population count of *V. aestuarianus*. Experiments were performed in quadruplicate (12 oysters tested per each substrate, Figure 1).

### Enumeration of culturable vibrios present in hemolymph

Hemolymph extracted from every single oyster was serially diluted in sterile PBS and spread onto TCBS agar plates. After 24 h incubation at 24°C, colonies were counted; results were expressed as colony‐forming units (CFU) per mL and represent an average of triplicate samples. For the identification of *V. aestuarianus*, DNA was extracted from selected morphotypes by boiling (10 min, 99°C) and analysed by species‐specific real‐time PCR assay (see Section 4.7).

### *Enumeration of total* Vibrio aestuarianus *and* Vibrio *spp. bacteria by real‐time PCR*

DNA was extracted from hemolymph of each single oyster using the High Pure PCR template preparation kit (Roche Diagnostics, Mannheim, Germany) according to the manufacturer's instructions. Real‐time PCR for total *V. aestuarianus* and *Vibrio* spp. cell counting was performed using LightCyler (Roche Diagnostics) with a Taqman or a SYBR Green real‐time PCR protocol, respectively. For *Vibrio* spp. enumeration, genus‐specific primers (F‐GGCGTAAAGCGCATGCAGGT; R‐GAAATTCTACCCCCCTCTACAG) (Thompson et al., 2004) were used following conditions described in Vezzulli, Pezzati, et al. (2015) and Vezzulli, Stauder, et al. (2015). Real‐time PCR for the enumeration of *V. aestuarianus* was performed using species‐specific primers and probe (DNAj F GTATGAAATTTTAACTGACCCACAA; DNAjR CAATTTCTTTCGAACAACCAC; DNAj probe FAM–TGGTAGCGCAGACTTCGGCGAC – BHQ2) (Saulnier et al., 2009) according to Vezzulli, Pezzati, et al. (2015) and Vezzulli, Stauder, et al. (2015). Accurately quantified copy number of genomic DNA of *V. aestuarianus* 02/041 was used as a standard. Results are expressed as an average of triplicate samples.

### Lysosomal membrane stability (LMS) test

LMS represents the most sensitive biomarker of stress at the cellular level. Measurements of LMS in animals from climatically and physically diverse ecosystems indicate that it is potentially a universal indicator of health status (OSPAR Commission, 2012). LMS was evaluated by the neutral red retention time (NRRT) assay as previously described and adapted for oysters (Balbi et al., 2013). Briefly, hemolymph from 3 to 5 oysters was pooled, hemocyte monolayers were prepared on glass slides, washed out and incubated with 20 μL of a neutral red (NR) solution. After 15 min, excess dye was washed out, 20 μL of ASW was added, and slides were sealed with a coverslip. Every 15 min, slides were examined under optical microscope and the percentage of cells showing loss of dye from lysosomes in each field was evaluated. For each time point, 10 fields were randomly observed, each containing 8–10 cells. End point of the assay was defined as the time at which 50% of the cells showed sign of lysosomal leaking, that is, the cytosol becoming red and the cells rounded. Triplicate preparations were performed for each sample. All incubations were carried out at 18°C. The data are reported as % of control group.

### Aminopeptidase fluorogenic assays

Association/incorporation of *V. aestuarianus* 02/041 bacteria with the different substrates were performed as previously described (Figure 1B). After 2 h incubation at 18°C, 4.5 mL of the suspensions including plankton vectors and associated bacteria were collected and used for aminopeptidase fluorogenic assays. In parallel, the same assay was performed with other bacterial strains cited above. Briefly, samples were added with 0.5 mL of L‐leucine‐4‐methylcoumarinyl‐7‐amide (Leu‐MCA, final concentration 500 μM), incubated for 1 h (enzymatic activity increased linearly with time up to 3 h) in the dark at 18°C. Enzymatic rates expressed as μmol/10^8^ cells/h were assessed by fluorometric analysis (at 380 nm excitation, 440 nm emission for Leu‐MCA) and corrected by subtracting the corresponding control values (Meyer‐Reil, 1986).

### Statistical analysis

The *t*‐test was used to compare differences in measured microbiological and biological variables between samples (treatment vs. control).

### RNA transcriptome analysis

The remaining suspension of *V. aestuarianus* 02/041 associated/non‐associated with marine aggregates (Figure 1B) was centrifuged (1000 g, 5 min), supernatants were kindly removed and the resulting pellets (e.g., bacteria only and marine aggregates plus bacteria), were used for total RNA extraction using the RNeasyPlus 96 kit from Qiagen. 10 μL of β‐mercaptoethanol (14.3 M) per mL of buffer RLTplus lysis was added before use. Concentration and purity of the total RNA extracted were assessed with RiboGreen (Quant‐iT™ RiboGreen® RNA Reagent and Kit, Invitrogen) and the Agilent 2100 Bioanalyzer system (total RNA Pico Chip for Prokaryotes; Agilent Technologies), respectively. Illumina's TruSeq stranded RNA library preparation kit including ribodepletion was used to construct libraries from total RNA. For Ribodepletion the ‘Ribo‐Zero® rRNA removal Kit (Bacteria)’ and library preparation the Illumina TruSeq Stranded mRNA Sample Preparation (Part # 15031047 Rev. E) were employed (Illumina, Inc. San Diego, USA). The libraries were quality controlled using a Quant‐iT RiboGreen RNA Assay Kit (Invitrogen Corporation, Carlsbad, CA) and the Agilent 2100 bioanalyzer ‘High Sensitivity DNA Chips’ (Agilent Technologies, Palo Alto, CA). The Illumina NextSeq 550 platform and a high‐output v2 kit (75 cycles) were used to sequence the libraries. The produced single‐end reads which passed Illumina's chastity filter were subject to de‐multiplexing and trimming of Illumina adaptor residuals using Illumina's bcl2fastq software version 2.19.1.403. The produced single‐end reads which passed Illumina's chastity filter were subject to de‐multiplexing and trimming of Illumina adaptor residuals using Illumina's bcl2fastq software v2.19.1.403 and Trimmomatic v0.33 (Bolger et al., 2014) and then mapped onto a *V. aestuarianus* 02/041 reference genome with Bowtie2 v2.4.5 (Langmead & Salzberg, 2012). Reads mappings to CDSs were counted with the software HT‐SEQ count v0.6.0 (Anders et al., 2015). Statistical analysis of differential expression on raw counts was conducted in R v4.2.2 (R Core Team, 2022) using the package DESeq2 v1.38.3 (Love et al., 2014) and visualized with ggplot2 (Wickham, 2016). Functional categories were assigned based on manual curation of the COG and GO annotations. RNA isolation, library preparation, sequencing and data quality analysis described in this section were performed by Microsynth AG (Balgach, Switzerland). Sequencing data were deposited in National Center for Biotechnology Information (NCBI) and can be accessed via BioProject ID PRJNA962710.

## AUTHOR CONTRIBUTIONS

**Lapo Doni:** Conceptualization (equal); data curation (equal); formal analysis (equal); investigation (equal); methodology (equal); software (lead); writing – original draft (lead). **Giovanni Tassitro:** Conceptualization (equal); data curation (equal); formal analysis (equal); investigation (equal); methodology (equal); writing – original draft (equal). **Caterina Oliveri:** Data curation (equal); formal analysis (equal); methodology (equal); writing – review and editing (equal). **Teresa Balbi:** Formal analysis (equal); methodology (equal); writing – review and editing (equal). **Manon Auguste:** Formal analysis (equal); methodology (equal); writing – review and editing (equal). **Alberto Pallavicini:** Formal analysis (equal); methodology (equal); writing – review and editing (equal). **Laura Canesi:** Formal analysis (equal); methodology (equal); writing – review and editing (equal). **Carla Pruzzo:** Conceptualization (lead); investigation (equal); methodology (equal); supervision (lead); writing – original draft (lead). **Luigi Vezzulli:** Conceptualization (lead); funding acquisition (lead); investigation (lead); methodology (equal); supervision (lead); writing – original draft (lead).

## CONFLICT OF INTEREST STATEMENT

The authors declare that the research was conducted in the absence of any commercial or financial relationships that could be construed as a potential conflict of interest.

## Supporting information


**Table S1.** Results of differential transcriptome analysis of *V. aestuarianus* 02/041 associated/non‐associated to marine aggregates.

## Data Availability

The authors confirm that the data supporting the findings of this study are available within the article and its supplementary materials. Sequencing data were deposited in National Center for Biotechnology Information (NCBI) and can be accessed via BioProject ID PRJNA962710.
